# Stress Sensors and Signal Transducers in Cyanobacteria

**DOI:** 10.3390/s100302386

**Published:** 2010-03-23

**Authors:** Dmitry A. Los, Anna Zorina, Maria Sinetova, Sergey Kryazhov, Kirill Mironov, Vladislav V. Zinchenko

**Affiliations:** 1 Laboratory of Intracellular Regulation, Institute of Plant Physiology, Russian Academy of Sciences, Botanicheskaya street 35, 127276, Moscow, Russia; E-Mails: tarlonc@yandex.ru (A.Z.); s_masha@list.ru (M.S.); kirill.mironoff@gmail.com (K.M.); 2 Department of Genetics, Faculty of Biology, Moscow State University, Moscow, Russia; E-Mails: seryik@rambler.ru (S.K.); zinchenko@mail.bio.msu.ru (V.V.Z.)

**Keywords:** histidine kinase, response regulator, sensor, serine-threonine kinase, stress, supercoiling, transducer

## Abstract

In living cells, the perception of environmental stress and the subsequent transduction of stress signals are primary events in the acclimation to changes in the environment. Some molecular sensors and transducers of environmental stress cannot be identified by traditional and conventional methods. Based on genomic information, a systematic approach has been applied to the solution of this problem in cyanobacteria, involving mutagenesis of potential sensors and signal transducers in combination with DNA microarray analyses for the genome-wide expression of genes. Forty-five genes for the histidine kinases (Hiks), 12 genes for serine-threonine protein kinases (Spks), 42 genes for response regulators (Rres), seven genes for RNA polymerase sigma factors, and nearly 70 genes for transcription factors have been successfully inactivated by targeted mutagenesis in the unicellular cyanobacterium *Synechocystis* sp. PCC 6803. Screening of mutant libraries by genome-wide DNA microarray analysis under various stress and non-stress conditions has allowed identification of proteins that perceive and transduce signals of environmental stress. Here we summarize recent progress in the identification of sensory and regulatory systems, including Hiks, Rres, Spks, sigma factors, transcription factors, and the role of genomic DNA supercoiling in the regulation of the responses of cyanobacterial cells to various types of stress.

## 1. Introduction

**Figure 1.**
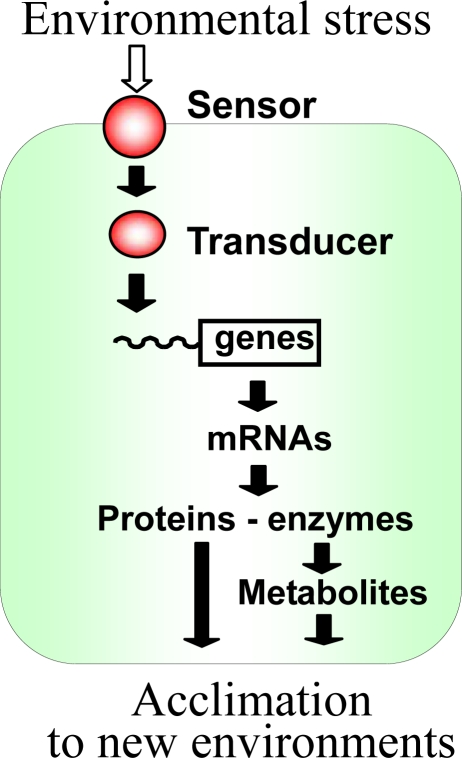
A general scheme showing the responses of a cyanobacterial cell to environmental stress. Adopted from [28]. Published with permission of Horizon Scientific Press / Caister Academic Press.

Environmental stresses influence the physiological activities of living organisms. When a change in the environment exceeds a certain threshold level, the activities of some enzymes are inhibited or abolished and those of others are enhanced or induced. In response to moderate stress, many organisms activate sets of genes that are specific to the individual type of stress. Specific proteins are synthesized and some of these proteins, in turn, participate in the synthesis of certain stress-specific metabolites. The proteins and metabolites that are synthesized de novo in response to stress are important for the acclimation of an organism and/or a cell to the new environment (Figure 1).

The primary steps in acclimation to environmental stress are the perception of such stress and transduction of the resulting signal. Organisms and/or individual cells are equipped with sensors and signal transducers that perceive and transduce signals from changing environment. They are mostly specific to individual types of environmental stress.

The unicellular cyanobacteria have several features that make them particularly suitable for studies of stress responses at the molecular level. The general features of the plasma and thylakoid membranes of cyanobacterial cells are similar to those of the chloroplasts of higher plants in terms of lipid composition and the assembly of membranes. Therefore, cyanobacteria can be expected to serve as powerful model systems for studying the molecular mechanisms of the responses and acclimation to stress [1,2], also these mechanisms may provide models that are applicable to higher plants as well.

Many strains of cyanobacteria, e.g., *Synechocystis* sp. PCC 6803 (hereafter, *Synechocystis*), are naturally competent. It means that foreign DNA may be incorporated into the cells integrated into their genomes by homologous recombination at high frequency [3,4]. As a result, cyanobacteria are widely used by researchers for the production of mutants with disrupted genes of interest [5,6].

Kaneko *et al*. determined the entire nucleotide sequence of the genome of *Synechocystis* [7] together with the entire sequences of four plasmids harbored by *Synechocystis* [8]. This is particularly useful as basic information, which can be exploited for genome-wide studies of gene expression. About 10 years ago, Takara Bio Co. (Ohtu, Japan) initiated the production of a genome-wide DNA microarrays for the analysis of gene expression in *Synechocystis*. The DNA microarray covers 3,079 (97%) of the 3,165 genes on the chromosome of *Synechocystis*, exluding 99 genes for transposases. It does not carry the genes from the four plasmids as well. The original results of analysis of patterns of gene expression in this cyanobacterium can be found in the KEGG expression database (Lists of experimental data are available at http://www.genome.jp/kegg/expression/).

In this review, we summarize recent progress in studies of sensors and signal transducers of environmental stress in *Synechocystis* that involved both systematic mutagenesis and the use of DNA microarrays.

## 2. Discussion

### 2.1. Potential Sensors and Signal Transducers in Cyanobacteria

The existence of two-component sensor-transducer systems has been well established in *Escherichia coli* [9] and *Bacillus subtilis* [10]. Each two-component system consists of a histidine kinase (Hik) and a cognate response regulator (Rre). In *E. coli* and *B. subtilis*, the genes for the two components of a single system are, in many cases, located close to one another on the chromosome. The Hik perceives a change in the environment via its sensor domain and then a conserved histidine residue within the histidine kinase domain is autophosphorylated, with ATP as the donor of the phosphate group [9]. The phosphate group is transferred from the Hik to a conserved aspartate residue in the receiver domain of the cognate transducer, Rre. Upon phosphorylation, the Rre changes its conformation, and this change allows the binding of the Rre to the promoter regions of genes that are located further downstream in the acclimation pathway (Figure 1).

In cyanobacteria, two-component systems, serine/threonine protein kinases (Spks), and sigma factors of RNA polymerase are conserved as potential candidates of sensors and transducers of environmental signals. Two-component systems have been found in prokaryotes (including cyanobacteria), fungi, yeasts, plants and lower animals [11], but not in higher animals. In eukaryotes and, in particular, in higher plants and higher animals, Spks or tyrosine protein kinases are the major sensors and/or transducers of environmental signals [12]. Sigma factors of RNA polymerases and regulatory factors that modify transcription are also involved in the regulation of gene expression. In addition, environmental stress can affect the superhelicity of DNA, which has been postulated to regulate gene expression [13,14].

### 2.2. Two-component Regulatory Systems in Perception and Transduction of Environmental Signals

The availability of the complete sequence of the *Synechocystis* genome [7] has allowed the construction of “knockout” libraries of specific sets of genes by targeted mutagenesis. This technique was used successfully for the identification of many potential sensors and transducers of environmental signals in *Synechocystis* [15–17]. In addition, DNA microarray analysis of the genome-wide expression of genes has allowed examination of the effects of mutations in specific genes for Hiks and Rres on the global expression of genes.

The *Synechocystis* chromosome includes 44 putative genes for Hiks [7,18] and there are three genes for Hiks on its plasmids [8]. There are 42 putative genes for Rres on the chromosome and three on the plasmids. These 47 Hiks and 45 Rres are candidates for sensors and transducers of environmental signals. The major difficulty in association of genes for Hiks with their respective cognate Rres in *Synechocystis* is that they are scattered on its genome, and as a rule, are not organized into operons, as they are in *E. coli* or *B. subtilis*. Thus, investigation of sensory Hiks and their cognate Rres requires mutations in individual Hik and Rre with subsequent examination of the stress-inducible expression of genes in the resultant mutants. In a series of experiments, 44 of the 47 *hik* genes and 42 of the 45 *rre* genes have been replaced with mutated genes (http://www.kazusa.or.jp/cyano/synechocystis/mutants/index.html). Several members of signal transduction pathway, which include *hik2, hik11, hik26, rre23, rre25* and *rre26* genes, were segregated incompletely.

### 2.3. Positive and Negative Regulation of Gene Expression

**Figure 2.**
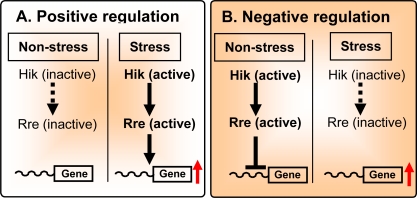
Schematic representation of positive (A) and negative (B) modes regulation of stress-inducible expression of genes. Solid arrows indicate signals that activate downstream components and dotted arrows indicate their absence. The inverted ‘T’ indicates signals that repress the expression of downstream genes. Red arrows correspond to the enhancement of gene expression. Adopted from [28]. Published with permission of Horizon Scientific Press / Caister Academic Press.

In priciple, two types of regulation of stress-inducible gene expression are mediated by Hik/Rre systems, namely, positive regulation and negative regulation (Figure 2). In positive regulation (Figure 2A), a Hik is inactive under non-stress conditions and, as a result, the corresponding Rre is inactive. Genes that are regulated by this type of a two-component system are silent under non-stress conditions.

In stressed cells, the Hik is activated by phosphorylation and then the signal is transferred to the cognate Rre, which enhances the expression of genes that are silent under non-stress conditions. Most of the stress-inducible regulation of gene expression in *Synechocystis* is associated with this type of regulation [19].

Negative regulation of the stress-inducible expression of genes implies that the Hik and its cognate Rre are active under non-stress conditions. As a result, they repress genes under non-stress conditions. In stressed cells, the Hik and Rre become inactive, resulting in expression of the previously repressed genes (Figure 2B).

Knockout mutation of either the Hik or the Rre in a two-component system for negative regulation has a marked effect on gene expression under non-stress conditions. The expression of genes that are controlled by a negatively regulating two-component system is enhanced under non-stress conditions. This allows relatively easy identification of the specific signal-transduction pathway controlled by a specific Hik and its cognate Rre. By contrast, knockout mutation of a Hik and Rre in a two-component system, that operates via positive regulation, does not result in any significant effects on gene expression under non-stress conditions. In this type of two-component system, the identification of the Hik and the Rre in a specific signal-transduction pathway requires the screening of knockout libraries of *hik* and *rre* genes under individual stress conditions.

Screening of the *hik* mutant library by examination of the genome-wide expression of genes with DNA microarrays revealed that mutation of the *hik20, hik27* and *hik34* genes induces significant changes in gene expression, in the absence of any change in environmental conditions (*i.e.,* in the absence of stress). Therefore, we postulated that the Hiks encoded by these genes might regulate gene expression in a negative manner. Mutation of the other 41 genes did not significantly alter gene expression, suggesting that Hiks encoded by these 41 genes regulate stress-inducible expression of genes in a positive manner. Although some authors have reported that Hik33 regulates gene expression in a negative manner, their conclusion might have arisen from the use of a mutant that harbored some additional mutation(s) [20]. Complementation experiments, with analysis of the genome-wide expression of genes, are necessary to evaluate this discrepancy and confirm this hypothesis.

### 2.4. Positive Regulation

#### 2.4.1. The Hik33-Rre26 System Controls the Expression of Cold-Inducible Genes

The histidine kinase Hik33 (Sll0698) has been identified as a cold sensor in *Synechocystis* [21]. This Hik has been earlier described as a component of the drug-resistance machinery DspA [22,23], and it is a homolog of NblS of *Synechococcus*, which may be involved in the regulation of genes that are induced under nitrogen limiting conditions [24]. However, the Hik33 does not seem to contribute significantly to the transduction of nutrient-related signals in *Synechocystis* [25].

**Figure 3.**
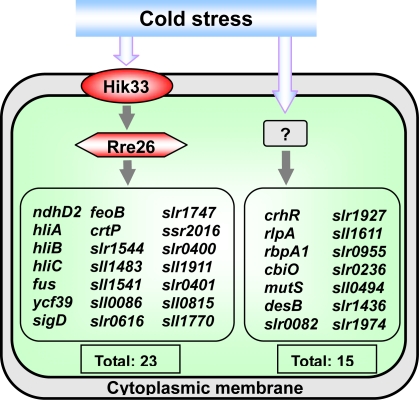
Schematic presentation of two-component system Hik33-Rre26 involved in the transduction of low-temperature stress. Primary signals are represented by open blue arrows. The sensory histidine kinase Hik33 supposedly perceives the temperature-induced rigidification in the cytoplasmic membrane. It tranduces the phosphoryl group to the response regulator Rre26, which itself might bind to promoter region of the genes to induce their transcription. Uncharacterized mechanisms are represented by question marks. Genes with induction factors (ratios of transcript levels of stressed cells to those of non-stressed cells) higher than 3:1 are included in these scheme. Adopted from [28]. Published with permission of Horizon Scientific Press / Caister Academic Press.

DNA microarray analysis of *hik33* mutant cells indicated that it regulates the expression of 23 of 38 highly cold-inducible genes (Figure 3). These 23 genes include *ndhD2, hliA, hliB, hliC*, *fus, feoB*, *crtP*, as well as genes for proteins of unknown function. Yet, 15 of the 38 cold-inducible genes were not regulated by Hik33. It was suggested that *Synechocystis* might have another pathway for transduction of the low-temperature signal (see section 2.6). The genes that are not controlled by Hik33 include highly cold-inducible genes, e.g., *crhR*, which encodes an RNA helicase and *rbp1*, which encodes a RNA-binding protein.

To identify the Rre that is located downstream of Hik33, an Rre knockout library was screened by RNA slot-blot hybridization using, as probes, some of the cold-inducible genes whose expression is controlled by Hik33. Rre26 has been identified as a candidate for the Rre that, with Hik33, constitutes a two-component system for cold-signal transduction [26]. Kappel and van Waarbergen [27] have demonstrated that Rre26 binds to the promoter region of the *hliB* gene, suggesting that this response regulator might be involved in the transduction of the low-temperature signal.

**Figure 4.**
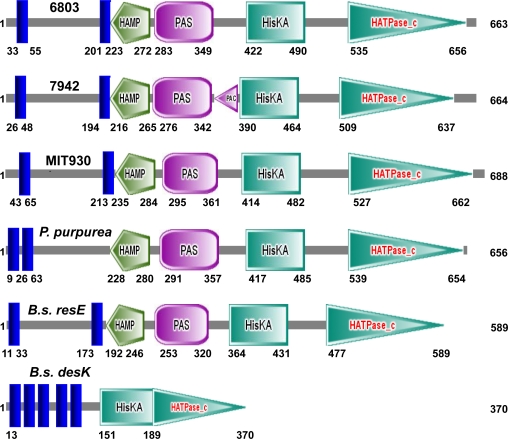
Schematic representation of the monomeric Hik33 of *Synechocystis* sp. PCC 6803 and its homologs from other organisms: 7942–*Synechococcus elongatus* PCC7942; MIT9301–*Prochlorococcus* strain MIT 9301; *P. purpurea*–*Porphyra purpurea; B.s.*–*Bacillus subtilis*. Numbers along the polypeptide sequences represent the corresponding numbers of amino acids starting from the first methionine. HAMP, PAS, PAC, HisKA, HATPase_c–the characteristic domains found by SMART software (http://smart.embl-heidelberg.de/). Hepcidin antimicrobial peptide (HAMP), HAMP-linker domains; LZ, leucine zipper domains; PER-ARNT-SIM (PAS), PAS domains that contain the amino acid motifs Per, Arnt, Sim and phytochrome [36]; blue rods represent the transmembrane domains.

It has been assumed for many years that the increased rigidity of membranes upon a downward shift in temperature should be the primary signal of cold stress. If this assumption is valid, how might Hik33 perceive such rigidification? The Hik33 sensory kinase includes two transmembrane domains, a HAMP linker, a leucine zipper, a PAS domain and a histidine kinase domain (Figure 4; [2,28,29]).

The HAMP linker contains two helical regions, in tandem, that are assumed to transduce stress signals across the membrane via intramolecular structural changes [30,31]. Hulko *et al*. [32] demonstrated recently that the HAMP linker domain is involved in the dimerization of a membrane-bound Hik and converts an extra- or intra-membrane signal to rotational movement of the Hik molecule. This movement activates the kinase domain [30]. In Hik33, the two transmembrane domains might sense changes in membrane rigidity since they are the segments of Hik33 that are associated with the lipid phase of the membrane [2,28,29]. Tasaka *et al*. [33] produced a series of *Synechocystis* mutants, in which the extent of unsaturation of fatty acids was modified in a step-wise manner. Changes in the fluidity of membrane lipids, due to each mutation, were verified by Fourier Transform Infrared spectroscopy (FTIR) [34,35]. Examination of the cold-induced expression of genes in these mutant cells with DNA microarrays revealed that rigidification of membrane lipids apparently enhanced the responses of gene expression to low temperature.

These various findings indicated that Hik33 regulates the expression of many genes. They also suggested that the activity of Hik33 in the sensing of low temperature depends on membrane rigidity and that there are at least two other cold sensors, one depending on membrane rigidness, while the other functions independently of the saturation of membrane lipids [29,34].

The homologs of Hik33 were found in the genomes of other organisms, including *Synechococcus elongatus* PCC 7942 (GeneBank Accession CP000100), several species of *Prochlorococcus marinus* (e.g., strain MIT 9301, GenBank Accession CP000576), chloroplast genome of red alga *Porphyra purpurea* (GenBank Accession U38804) (Figure 4).

The cold-sensing and membrane fluidity sensing histidine kinase, DesK, was also identified in *Bacillus subtilis* [37,38]. DesK transfers the phosphate group to its cognate response regulator, DesR [39,40], which controls transcription of the gene for the Δ5-desaturase [41,42].

Complementation of the deletion of Hik33 in *Synechocystis*, revealed that the analogous sequence of *Synechococcus elongatus* can efficiently substitute for the cold-sensor, while the corresponding sequences of *P. purpurea* or *B. subtilis* cannot [43]. This might be due to the differences in organization of the transmembrane domains in the sensor proteins (Figure 4). In *B. subtilis*, however, there is another structural homolog of Hik33, the histidine kinase ResE [44,45], which is involved in nitrogen regulation under oxygen limiting conditions. Its involvement in cold-sensing and the possibility to substitute for Hik33 was never studied.

#### 2.4.2. Five Two-Component Systems Participate in the Perception and Transduction of Salt-Stress and Hyperosmotic-Stress Signals

In the literature, hyperosmotic stress and salt stress are frequently regarded as identical forms of stress. However, they are distinctly different. Hyperosmotic stress causes the efflux of water from the cytoplasm, resulting in a decrease in cytoplasmic volume and increased concentrations of cytoplasmic solutes and other components. By contrast, salt stress decreases the cytoplasmic volume only transiently and to a small extent [46], while rapidly increasing cytoplasmic concentrations of Na^+^ and Cl^−^ ions [47] *via* an influx of NaCl through Na^+^/K^+^ and Cl^−^ channels.

Comprehensive analysis with DNA microarrays, of the *hik* mutant library upon exposure of the cells to salt (NaCl) stress revealed that the inducibility of gene expression by elevated levels of NaCl was significantly affected in Δ*hik16* (*slr1805*), Δ*hik33* (*sll0698*), Δ*hik34* (*slr1285*), and Δ*hik41* (*sll1229*) mutant cells [48]. In each of these mutants, the expression of several genes was no longer induced by salt or the extent of inducibility by salt was markedly reduced.

**Figure 5.**
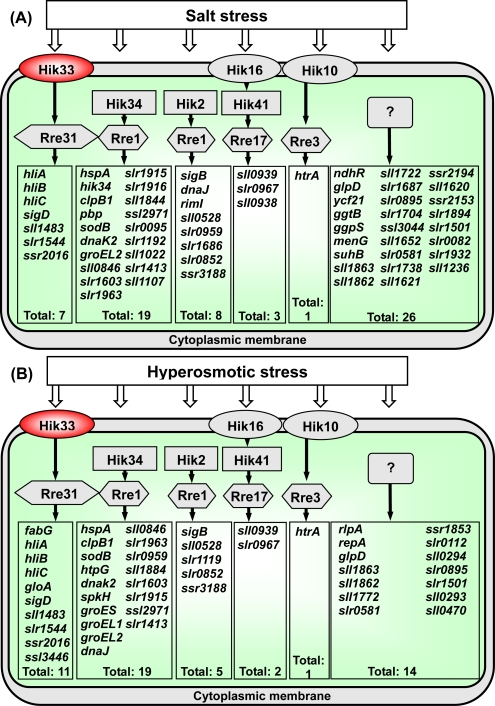
Hypothetical schemes of two-component systems that are involved in the transduction of salt stress (A) and hyperosmotic stress (B), as well as the genes that are under the control of the individual two-component systems. Primary signals are represented by open arrows. Hiks that posess transmembrane domains are indicated as ellipses (Hik33, Hik16, Hik10); Hik33 is in a red ellipse; soluble Hiks are shown as horizontal boxes (Hik34, Hik2, Hik41); Rres are indicated as hexagons, and selectively regulated genes are shown in vertical boxes. Uncharacterized mechanisms are represented by question marks. Genes with induction factors higher than 4.0 are included in these schemes. Adopted from [28]. Published with permission of Horizon Scientific Press / Caister Academic Press.

Five Hik-Rre systems, namely, Hik33-Rre31, Hik10-Rre3, Hik16-Hik41-Rre17, Hik2-Rre1 and Hik34-Rre1, have been found that are involved in the perception of salt stress and transduction of the signal [17]. Figure 5A shows a hypothetical scheme for the salt signal-transducing systems that involve these Hiks and Rres. The scheme includes those salt-inducible genes that are controlled by individual Hik-Rre two-component systems.

The perception and transduction of hyperosmotic-stress signals involve the same five Hik-Rre systems. Figure 5B shows the signal-transduction pathways that operate when cells are exposed to hyperosmotic stress, as well as the hyperosmotic stress-inducible genes whose expression is controlled by the individual Hik-Rre systems [16,17].

As shown schematically in Figure 5, hyperosmotic stress and salt stress, respectively, appear to be perceived by identical sets of Hik-Rre systems in *Synechocystis*, which regulate rather similar sets of genes, albeit to different extents.

However, it is also clear that identical Hik-Rre systems may also control the expression of different sets of genes under hyperosmotic stress and salt stress [16]. Typical examples are the Hik33-Rre31 and Hik34-Rre1 pairs mentioned above. The Hik33-Rre31 two-component system regulates the expression of *fabG* and *gloA* as hyperosmotic stress-specific genes and that of *pgr5, nblA1* and *nblA2* as oxidative stress-specific genes.

The Hik34-Rre1 system regulates the expression of *sll1107* as a salt stress-specific gene and that of *htpG* as a hyperosmotic stress-specific gene (Figure 5).

The current concept of two-component systems does not explain how the Hik33-Rre31 system controls the differential expression of the two different sets of genes under different types of stress. It seems reasonable to assume the existance of some factor(s) that provide each two-component system with the strict specificity that is related to the specific nature of the stress. The example of such factor might be a small polipeptide SipA (Ssl3451), which enhances the activity of Hik33 *in vivo* and *in vitro* [49].

#### 2.4.3. Histidine Kinases That are Involved in Perception of Oxidative Stress and Light

Four histidine kinases, Hik34, Hik16, Hik41, and Hik33, regulate the oxidative stress-inducible expression of genes [20]. Hik34, which was identified as a sensor/transducer of signals due to heat, salt, and hyperosmotic stress, also regulates the expression of the H_2_O_2_-inducible *htpG* gene. Hik16 and Hik41 together regulate the expression of *sll0967* and *sll0939*. Hik33 is the main contributor to the regulation of H_2_O_2_-inducible gene expression and regulates the induction of expression of 22 of the 26 H_2_O_2_-inducible genes that are under the control of histidine kinases. Hik33 regulates the expression of the *ndhD2* gene, *hli* genes, *pgr5* gene (for ferredoxin plastoquinone reductase), *nblA1* and *nblA2* genes (for proteins involved in the degradation of phycobilisomes), and of several other genes. The cognate Rre of each of these Hiks in the transduction of oxidative signals has not yet been identified [20].

The potential sensors of light in *Synechocystis* are Histidine Kinases with phytochrome-like features [50,51]. The *Synechocystis* genome contains six *hik* genes, namely, *hik35* (*slr0473*), *hik3* (*sll1124*), *hik32* (*sll1473/sll1475*), *hik1* (*slr1393), hik44* (*slr1212*)*,* and *hik24* (*slr1969*), that are homologous by various degrees to plant genes for phytochromes.

When the *hik35* (*cph1*) gene was expressed in *E. coli* cells that synthesized phycocyanobilin, the *E. coli* cells produced an adduct with a red/far-red photoreversible signature that is typical of phytochrome [52]. The *rre27* (*rcp1* or *slr0474*) gene is located adjacent to the *hik35* gene [53]. Insertional inactivation of *hik35* in *Synechocystis* impaired the growth of mutant cells under continuous far-red light [54].

An attempt to identify the molecular targets of Hik35 (Cph1) activity was made using DNA microarray [55]. In wild-type and two lines of phytochrome-mutant cells, 25% of all 3,165 putative genes responded to light. Red light predominantly enhanced the expression of genes whose products are involved in transcription, translation, and photosynthesis, whereas far-red light raised the levels of transcripts of genes whose expression is induced by various kinds of stress. The mutation of *hik35* (*cph1*) altered the light-dependent expression of approximately 20 genes. Hence, light receptor(s) different from this two-component system might trigger global red/far-red-induced alterations in the profile of gene expression.

It seems unlikely that all Hiks mentioned above are typical phytochromes in terms of red/far-red reversibility of their activities. Indeed, Hik3 seems to be involved in blue light-dependent growth [56], and Hik44 (Etr1) binds ethylene [57]. The potential involvement in the perception of light-stress signals of Hik32 [which is, incidentally, intact in a strain from the Pasteur Culture Collection but is disrupted by an insertion in a strain from Dupont [58], Hik1, and Hik24 merits further investigation. The involvement of the Hik33-Rre26 system in light-regulated gene expression has also been suggested [59,60].

In *Synechococcus elongatus* PCC 7942, two histidine kinases, namely, SasA (an ortholog of Hik8) and CikA (an ortholog of Hik24), have been identified as signal transducers in the establishment of circadian rhythm [61–64]. SasA transfers a phosphate group to its cognate response regulator, RpaA (an ortholog of Rre31), in the presence of KaiC, and acts as a major mediator of circadian timing that regulates the oscillation of gene expression [62].

In the filamentous cyanobacterium *Calothrix* sp. PCC7601, CphA-RcpA and CphB-RcpB have been identified as two-component, light-sensing, phytochrome-like systems [65]. Both of these regulatory systems are homologous to Hik35-Rre27 in *Synechocystis*. In *Anabaena* a cyanobacterial rhodopsin was identified, whose biochemical and biophysical characteristics suggest its involvment in the regulation of chromatic adaptation [66].

#### 2.4.4. The Hik7-Rre29 System Controls Gene Expression during Phosphate Limitation

The *hik7* (*sll0337* or *sphS*) and *rre29* (*slr0081* or *sphR*) genes in *Synechocystis* encode proteins that are homologous, respectively, to PhoR and PhoB of *E. coli*, PhoR and PhoP of *B. subtilis*, and to SphS and SphR of *Synechococcus* sp. PCC 7942 [67]. The Hik7-Rre29 two-component system induces the expression of the *phoA* gene for alkaline phosphatase in response to phosphate limitation.

DNA microarray analysis revealed that the expression of 12 genes was strongly induced while that of one gene was strongly repressed when the supply of phosphate was limited [68]. The expression of all phosphate limitation-inducible genes was completely eliminated upon inactivation of either Hik7 or Rre29. The response regulator Rre29 binds to the upstream flanking regions of three genes at repetitive PyTTAAPyPy(T/A)-like sequences (where Py represents a pyrimidine). It was suggested that the Hik7-Rre29 two-component system might be the only system for the perception and transduction of the phosphate-limitation signal in *Synechocystis* [68].

However, a recent investigation demonstrated that another component, transcriptional regulator SphU (*slr0741*), is involved in transduction of the phosphate-limitation signal in *Synechocystis* [69,70]. This component negatively regulates the expression of the *phoA* gene for alkaline phosphatase. The threonine residue at position 167, adjacent to the histidine residue of SphS (Hik7) that can be phosphorylated in response to phosphate limitation, is important in the negative regulation mediated by SphU.

#### 2.4.5. The Hik30-Rre33 Signaling System for Excess of Nickel Ions

The Hik30-Rre33 (or NrsS-NrsR) two-component system regulates transcription of the *nrsBACD* operon, which is involved in the resistance to excess of Ni^2+^ ions [71]. Knockout mutation of either the *hik30* or the *rre33* gene abolishes the Ni^2+^-induced transcription of the *nrsBACD* operon, which encodes ABC-type Ni^2+^ ion transporter. In addition, mutation of Rre33 caused the accumulation of transcripts of genes for components of PSII, such as *psbA* and *psbD,* and suppressed that of genes in phycobilisomal operons, such as *apcAB* and *cpcBA,* under conditions of normal light intensity [72]. These observations suggested that the mutant cells might not be able to sense the accumulation of excess Ni^2+^ ions, which might have toxic effects on the cells. As a result, the mutant cells fail to survive when the concentration of Ni^2+^ ions in the growth medium is elevated. The possible mechanism of Ni^2+^ sensing implies that binding of a Ni^2+^ ion to the Hik30 causes its autophosphorylation. The phosphate group is then transferred to Rre33, which binds to the intergenic regions of *nrsRS* and *nrsBACD*, which are located close to each other on the chromosome, stimulating transcription of the latter operon. This example represents, therefore, a positive type of regulatory system.

### 2.5. Negative Regulation

#### 2.5.1. The Hik27-Rre16 Signaling System for Manganese Limitation

Hik27 (*slr0640*) and Rre16 (*slr1837*) constitute a two-component system for the perception of manganese limitation and transduction of the resultant signal [73]. This system regulates the expression of three genes, namely, *mntC, mntA*, and *mntB,* which constitute the *mntCAB* operon that encodes subunits of the ABC-type Mn^2+^ transporter [74,75].

**Figure 6.**
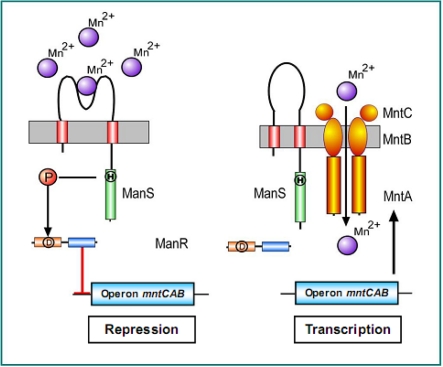
Hypothetical model for the sensing of Mn^2+^ ions, the transduction of the Mn^2+^ signal, and the regulation of expression of the *mntCAB* eperon that encodes the Mn^2+^ transporter. His and Asp residues that might be involved in a phosphorelay are indicated by the encircled H and D, respectively. Adopted from [73].

Hik27-Rre16 pair is active under non-stress conditions and represses the expression of the *mntCAB* operon according to a negative scheme of regulation provided in Figure 2B. Under normal conditions (enough Mn^2+^ in the growth medium) Mn^2+^ sensor, ManS, binds manganese ions in the outer medium and transfers the phosphoryl group to ManR, which, in its phosphorylated form, represses the *mntCAB* operon (Figure 6A). Under Mn^2+^ limitation, ManS is no longer phosphorylated, and unphosporylated ManR cannot bind to the promoter of *mntCAB* (Figure 6B).

The genes for the specific ABC-transporter are expressed to compensate the intracellular loss of Mn^2+^. Mutations in either *hik27* or *rre16* abolished the Mn^2+^ limitation-induced expression of the *mntCAB* operon. These mutation also allowed relatively easy identification of the MasS-ManR regulatory pair, since only *mntC, mntA*, and *mntB* genes were induced in the mutants ander normal growth conditions [73].

#### 2.5.2. Hik34 Controls Heat-stress Response

The histidine kinase, Hik34, was identified as an important contributor to the regulation of expression of heat-shock genes and acquisition of thermotolerance in *Synechocystis* [76]. Mutation of the *hik34* gene enhanced the levels of transcripts of a number of heat-shock genes, which included *htpG* and *groESL1*. Furthermore, overexpression of the *hik34* gene repressed the expression of these heat-shock genes. In addition, Hik34-mutant cells survived incubation at 48 °C for 3 h, while wild-type cells, and cells with mutations in other Hiks, failed to do so. However, mutation of the *hik34* gene had only an insignificant effect on the global expression of genes during incubation of the mutant cells at 44 °C for 20 min. Among 59 heat stress-inducible genes with ratios of transcript levels greater than 3:1, mutation of the *hik34* gene markedly decreased the heat inducibility of only two genes, namely, *htpG* and *slr1963*. Recombinant Hik34 expressed in *E. coli* was autophosphorylated *in vitro* at physiological temperatures but not at elevated temperatures, such as 44 °C [76]. Thus, it seems likely that Hik34 is involved in the negative regulation of the expression of certain heat-shock genes that might be related to thermotolerance in *Synechocystis*.

### 2.6. Other Potential Sensors and Transducers of Environmental Signals

#### 2.6.1. Serine/Threonine Protein Kinases and Protein Phosphatases

While eubacteria, including cyanobacteria, use two-component systems for many types of signal transduction, eukaryotes exploit Spk, Tyr kinases and protein phosphatases for similar purposes. The presence of genes for Ser/Thr kinases and phosphatases in cyanobacteria was revealed only when the complete sequence of the *Synechocystis* genome became available [77]. The putative proteins were identified by comparison of deduced amino acid sequences encoded by open-reading frames with known amino acid sequences of eukaryotic protein kinases.

Among 12 putative genes for Ser/Thr kinases in *Synechocystis*, seven encode proteins that belong to the PKN2 subfamily of Spks and five encode proteins that belong to the ABC1 subfamily of Spks [78]. The genes for kinases of the PKN2 type are designated *spkA, spkB, spkC, spkD, spkE, spkF* and *spkG* and those for kinases of the ABC1 type are designated *spkH, spkI, spkJ, spkK* and *spkL* (CyanoBase; http://www.kazusa.or.jp/cyano/). The functions of the products of only three of these genes have been characterized to date [79]. SpkA and SpkB appear to be involved in the control of cell motility [80,81], while SpkE is probably involved in the regulation of nitrogen metabolism [82].

A recent study with DNA microarrays demonstrated the relationships among the activity of SpkA, the genome-wide expression of certain genes, the formation of thick pili, and cell motility [83]. It is likely that, under non-stress conditions, SpkA activates the expression of the putative *pilA9*-*pilA10*-*pilA11*-*slr2018* operon and inactivates the expression of the *pilA5*-*pilA6* and *pilA1*-*pilA2* operons. Electron microscopy revealed that SpkA activity is essential for the formation of thick pili. It seems likely that SpkA is a regulator of the expression of these putative operons, whose products are regulators of the formation of thick pili, which are, in turn, essential for cell motility. The molecular mechanism responsible for the contribution of SpkB to cell motility remains to be clarified.

Protein phosphatases are also important participants in phosphorylation/dephosphorylation regulatory cascades. In *Synechocystis* signal transduction protein P(II) is dephosphorylated by protein phosphatase PphA (*sll1771*). Mutants lacking either PphA or P(II) were impaired in efficient utilization of nitrate as the nitrogen source [84,85].

Two protein-serine/threonine phosphatases Sll1033 (SynPPM3) and Sll1387 (SynPPP1) are homologues to the PPM and PPP families of phosphatases. The Sll1033 dephosphorylated phosphotyrosine- as well as phosphoserine-containing proteins *in vitro* with the preference to the latter. The Sll1387 dephosphorylated phosphoserine-containing proteins with comparable efficiencies [86]. Other putative Ser/Thr phosphatases are encoded by the following ORFs: *sll1365, slr0114, slr0328, slr1860, slr1983, slr2031* [87]. Further studies are necessary to reveal their function and to determine the potential candidates for the histidine kinase phosphatases.

#### 2.6.2. RNA Polymerase Sigma Factors

The initiation of transcription, mediated by RNA polymerase holoenzyme, is the main determinant for gene regulation in eubacteria. The eubacterial RNA polymerase holoenzyme is composed of the core enzyme with the subunit composition of α_2_, β, β′, ω, and a σ factor. In cyanobacteria, the β′ subunit is split into two parts and the N-terminal part has been named the γ subunit [88].

The core enzyme exhibits the RNA polymerase activity, while the σ factor is responsible for the recognition of a promoter sequence. Most bacteria synthesize several σ factors, each of them recognizing a unique set of promoter regions. Environmental conditions or developmental signals often cause major changes in gene expression by inducing a swap of σ factors. Two families of bacterial σ factors (σ^70^ and σ^54^) have been identified on the basis of structural and functional similarity. The σ^70^ family is further divided into three subgroups.

Group 1 is composed of primary σ factors (PSF) that are essential for cell viability and are mainly responsible for the transcription of genes expressed during the exponential-growth phase.

Those σ factors that show extensive amino acid similarity to PSF but are not essential for exponential growth belong to group 2. Cyanobacteria contain many group 2 σ factors, but the physiological roles of these numerous PSF-like σ factors are still unclear. Group 3 consists of σ factors that vary more considerably in amino acid sequence than those of groups 1 or 2. These so-called alternative σ factors are involved in the transcription of specific regulons expressed in extracytoplasmic stress conditions, during sporulation, or in the synthesis of flagella.

Nine open reading frames encode putative σ factors in the cyanobacterium *Synechocystis* [89,90]. According to sequence homology, the *sigA* gene encodes the PSF; the *sigB*, *sigC*, *sigD*, and *sigE* genes encode group 2 σ factors; and the *sigF*, *sigG*, *sigH*, and *sigI* genes encode group 3 σ factors. The attempts to characterize the functional role of each σ factor have been recently made.

Transcription of the ***sigA*** gene (*slr0653*) was the highest during the exponential phase of growth of *Synechocystis* cells and it was severely inhibited by limitation of light (or by the inhibitors of PSII), heat shock (42 °C), and salt stress (0.5 M NaCl) [91]. These findings together with the positive correlation between the amount of *sigA* mRNA and the growth rate of the cells, supports the hypothesis that the *sigA* gene encodes the PSF in *Synechocystis*.

Only tiny amounts of ***sigB*** (*sll0306*) transcripts were measured under standard growth conditions, and *sigB* transcripts totally disappeared after a long dark treatment. Changes in many environmental conditions (inhibition of the activity of PSII, heat, and salt stress) rapidly induce the transient increase of *sigB* mRNA. The involvement of the SigB in the control of transcription of the heat-shock genes and on acquired thermotolerance had been demonstrated in the mutant that lacks the corresponding gene [92].

The level of transcription of the ***sigC*** gene (*sll0184*) is small in optimal and stress conditions tested. SigC is supposedly involved in transcription of the specific genes during nitrogen starvation [93,94].

The ***sigE*** gene (*sll1689*) also produces very small amounts of transcripts in optimal and stress conditions. It was reported that SigE participates in positive regulation of sugar catabolic pathways [95,96].

The ***sigD*** (*sll2012*) is the only *sig* gene that produced moderate amounts of transcripts in the dark. Its expression was shown to be induced by DCMU, and it was not affected by any of stress treatments [91]. The exact function of SigD remains to be clarified. It was shown that SigB and SigD with another group 2 sigma, SigC, contribute to transcription for a subset of dark/light-responsive genes. Transcription of the sigD gene is significantly upregulated by high light and SigD recognizes the promoter of *psbA* that encodes the major protein D1 of the PSII [97–99]. Strong induction of *sigD* by H_2_O_2_ has been also demonstrated suggesting that SigD could play an important role in peroxide protection [100].

Some reports suggest the existence of a complicated crass-talk between the group 2 σ factors. These subunits are supposed to substitute each other during the growth (circadian regulation) and stress conditions [94,99,101].

The functional significance of the alternative σ factors that belong to group 3 have been accessed with the use of the mutants lacking *sigF*, *sigG*, and *sigH* genes [102,103].

The completely segregated ***sigF*** (*slr1564*) mutant of *Synechocystis* exhibited a pronounced defect in salt-stress-induced gene expression. Long term application of high-salt stress clearly diminished the survival rate of the SigF mutant in comparison to WT cells. The specific effect of salt, demonstrated in the phenotype of the SigF mutant, provides evidence that SigF represents a terminal element of a signal-transducing pathway sensing salt [102]. This pathway apparently targets stress proteins and proteins involved in the synthesis of pili, required for light-induced mobility of *Synechocystis* [104]. Recently, it was found that SigF autoregulates its own transcription and recognizes the promoter of *pilA1* that acts in pilus formation and motility. The *pilA1* promoter was recognized only by SigF and not by other sigma-factors in *Synechocystis* [103].

The mutant of *Synechocystis* deficient in **SigH** (*sll0856*) was able to tolerate all growth and stress conditions. The *sigH* transcript increased under heat stress. However, compared to the induction of the typical heat-shock genes *groEL*, the induction of *sigH* occurred rather late making it unlikely that the alternative σ factor SigH is responsible for the regulation of these heat-shock genes [102].

Only the σ factor **SigG** (*slr1545*) was found to be essential for growth of *Synechocystis* cells under optimal conditions, since the attempts to mutagenize the *sigG* locus resulted in merodiploids. In accordance with a possible involvement of SigG in transcription under standard growth conditions, the highest mRNA level of this gene was found in cells grown under these conditions. The mutant in SigG, however, demonstrated reduced survival time of cells under high-light conditions. In photosynthetic cells, high light intensities induce oxidative stress and it is intriguing to know whether any of cyanobacterial σ factors are involved in response to oxidative stress [102].

The remaining member of the group 3 family of σ factors, **SigI** (*sll0687*), has not yet being functionally characterized.

#### 2.6.3. Transcription Factors

**Figure 7.**
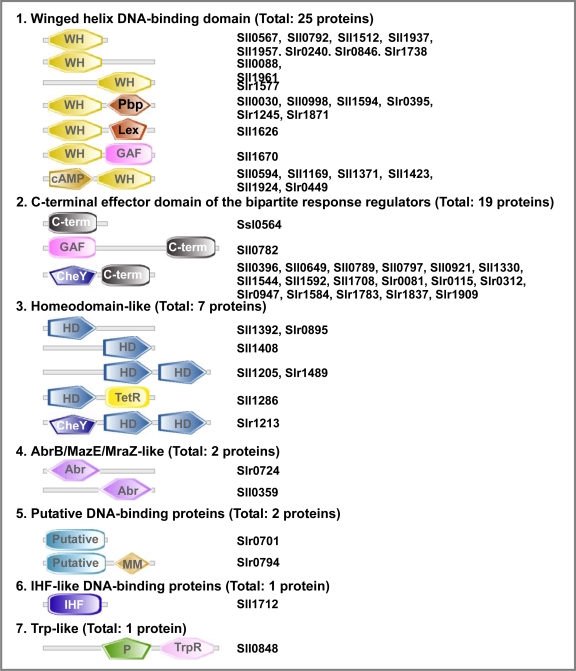
Transcription factors of *Synechocystis* classified according to the specific architecture of their DNA-binding domais. **WH**-“Winged helix” DNA-binding domain; **C-term**-C-terminal effector domain of the bipartite response regulators; **Abr**-AbrB/MazE/MraZ-like domain; Putative-Putative DNA-binding domain; **IHF**-IHF-like DNA-binding proteins; **HD**-Homeodomain-like; **TRP**-TrpR-like; **Pbp**-Periplasmic binding protein-like II; **GAF**-GAF domain; **cAMP**-cAMP-binding domain-like; **Lex**-LexA/Signal peptidase; **MM**-Precorrin-8X metylmutase; **P**-P-loop containing nucleotide triphosphate hydrolases; **TetR**-Tetracyclin repressor-like, **C**-terminal domain; **CheY**-CheY-like domain.

A search for and classification of DNA-binding transcription factors (TFs) in *Synechocystis*, was conducted using the sequence of the genome and a set of bioinformatic tools. Fifty-seven genes for TFs, that account for 1.7% of all genes, in the *Synechocystis* genome were found. The TFs include the DNA-binding domains of seven families (Figure 7).

The identified TFs contain the DNA-binding domains that could be subdivided into 7 groups: winged helix; C-terminal effector domain of the bipartite response regulators; homeodomain-like; AbrB/MazE/MraZ-like; putative DNA-binding; IHF-like DNA-binding proteins, and TrpR-like containing domains. With the exception of the AbrB/MazE/MraZ-like family, all the proteins contain «helix-turn-helix» motif characteristic for the DNA-binding proteins of prokaryotes [105]. The proteins of the AbrB/MazE/MraZ-like family are characterized by the presence of a newly discovered DNA-binding domain called «looped-hinge helix» [106].

Only 19% of TFs carry a single DNA-binding domain. Four proteins contain additional DNA-binding domain. The overwhelming majority of TFs contain two or more additional domains, which belong to one of three functional groups: enzymatic, small-molecule-binding, CheY-like domains. Enzymatic domain is present in 3 TFs. Small-molecule-binding domains are present in 14 proteins. The latter indicates that these TFs might be regulated by small molecules, such as cyclic NTPs and others. CheY-like domains, which are characteristic for the family of response regulators, were found in 18 representatives of TFs. Thus, these proteins might be regulated by phosphorylation. These observations suggest that the majority of transcription factors are regulated not only at the level of transcription, but also at a post-translational level.

The functions of several TFs have been characterized. The role of a LysR-type regulator of transcription, NdhR (Sll1594), was studied systematically with DNA microarrays [107]. NdhR negatively regulates the expression of its own gene, as well as that of the *ndhF3* and *ndhD3* genes for NDH-1 (NADH dehydrogenase-1) complex. NdhR also regulates the expression of the *nhaS1* (*slr1727*) gene for one of the Na^+^/H^+^ antiporters.

HrcA (Sll1670) encodes an ortholog of a protein that negatively regulates the expression of the heat-shock *grpE-dnaK-dnaJ* and *groESL* operons for chaperonins in *B. subtilis* [108]. In *Synechocystis*, HrcA regulates the expression of few so called heat-shock genes including *groESL* and *groEL2* [109]. Its interaction with other regulatiory systems, e.g., RNA polymerase sigma factors SigB, SigE, and/or sensory histidine kinase Hik34, has been suggested [110].

SufR (Sll0088) functions both as a transcriptional repressor of the *sufBCDS*, which is involved in the biogenesis of the iron-sulfur cluster in Photosystem-I, and as an autoregulator of *sufR* [111,112].

ArsR (Sll1957) represses the expression of the *arsBHC* operon, which consists of three genes: the *arsB* gene that encodes a putative arsenite and antimonite carrier; the *arsH* gene that encodes a protein of unknown function; and the *arsC* gene that encodes a putative arsenate reductase that confers arsenic resistance [113].

PerR (Slr1738) regulates a set of genes, which are induced in response to hydrogen peroxide [100]. It is also involved in reprogramming of cellular metabolism in response to excess cadmium concentrations [114]. Another TF, which is involved in regulation of response to oxidative stress is the autorepressor PrqR (Sll0886), which negatively regulates the prqR-prqA operon and the response to oxidative stress inductor methyl viologen [115]. Surprisingly, the point mutation in the DNA-binding domain of PrqR have lead to a reversed (changed from positive to negative) phototaxis induced with daylight and red light of low intensity, whereas the complete deletion of the *prqA* did not affect cell motility and type of phototaxis [116]. This suggests that the specificity of the regulator protein was changed in cells carrying the point *prqRL17Q* mutation. The latter mutation caused a decrease in transcription of gene *taxD1* for the photoreceptor of red light that controls positive phototaxis [117]. These data imply that mutation *prqRL17Q* changes the specificity of the PrqR repressor protein and thereby affects the regulation of phototaxis at the level of photoperception and signal transduction in cells.

The AbrB-like TF, Sll0359, is an activator of the expression of the operon *hoxEFUYH*, which encodes the bidirectional Ni-Fe hydrogenase [118,119].

The results of genome-wide analysis of transcription in the mutant defective in Sll1961, revealed that the TF Sll1961 is associated with modulation of photosystem stoichiometry (the ratio of two photosynthetic reaction centers-photosystem I and photosystem II) during acclimation to high light [120]. LuxR-type TF PedR (Ssl0564) is constitutively expressed in cells, and it senses the activity of photosynthetic electron transport [121]. When the activity of photosynthetic electron transport is low, PedR up-regulates the expression of *chlL*, *chlN*, *chlB*, and *slr1957* and down-regulates the *ndhD2*, *rpe*, and the *pedR*-sll0296 operon. When the photon flux density is elevated, and the supply of reducing equivalents from photosynthetic electron transport chain increases, the PedR is inactivated through its conformational change within 5 min [121]. This mechanism enables transient induction or repression of the target genes in response to changes in illumination.

### 2.7. DNA Supercoiling is Involved in the Perception of Stress Signals and the Regulation of Gene Expression

Alterations in the supercoiling of genomic DNA play important roles in the regulation of gene expression in response to environmental stress both in Gram-negative and Gram-positive bacteria [122–124]. It has been proposed that temperature-dependent alterations in DNA supercoiling might be one of the sensory mechanisms that regulate the expression of genes involved in the acclimation to low temperature [13,14]. Salt stress and hyperosmotic stress also affect the negative supercoiling of DNA and regulate gene transcription [125–127].

Studies of changes in the supercoiling of DNA were initially limited to plasmid DNAs in *E. coli*, *B. subtilis* and *Salmonella typhimurium*. Therefore, changes in gene expression due to changes in the supercoiling of chromosomal DNA, have mainly been assumed on the basis of changes in the linking numbers of plasmids [128–130]. An inhibitor of DNA gyrase, novobiocin [14,131], has been used to examine the effects of changes in the negative supercoiling of DNA on the genome-wide expression of genes in *Synechocystis* in response to cold stress. Novobiocin interacts with the ATP-binding site of the B-subunit of DNA gyrase. Cold stress caused an increase in the negative supercoiling of the promoter region of the *desB* gene for a fatty acid desaturase and directly controlled its expression at low temperatures [13,14], pointing that temperature-induced changes in supercoiling of DNA might contribute to stress-induced gene expression in cyanobacteria.

Recently, we applied DNA microarray-based analysis of gene expression and demonstrated that novobiocin, which inhibits stress-induced changes in DNA supercoiling, regulated the transcription of many genes that are involved in stress responses including the genes that are obligatory for acclimatization of cells to changed environments.

The function of the two-component regulatory systems, which are known as sensors and transducers of salt, cold, and heat stress (in particular, Hik33 and Hik34 histidine kinases), depends on the degree of supercoiling of the genomic DNA [13]. Thus, DNA supercoiling might regulate transcription of stress-inducible genes directly, and/or provide a permissive background for regulatory proteins, which switch on or off the expression of the downstream genes and ensure successful acclimatization of cells to stress conditions.

**Figure 8.**
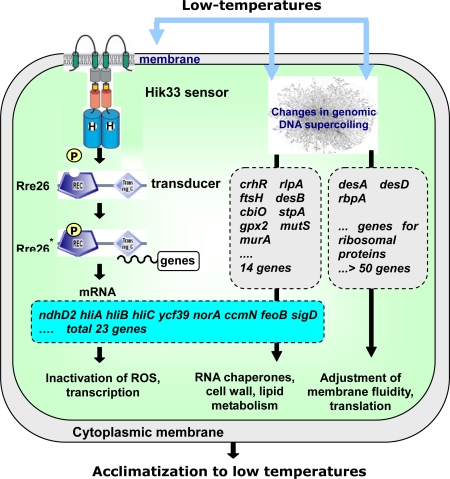
A scheme of the perception and transduction of cold-stress signals by cyanobacterial cells. The sensory histidine kinase Hik33 perceives the temperature-induced rigidification in the cytoplasmic membrane. Upon autophosphorylation of the Hik33 dimer, it tranduces the phosphoryl group to the response regulator Rre26, which binds to promoter region of the genes to induce their transcription. The Hik33-Rre26 two-component sensor and transduction system regulates a part of cold-inducible genes. This part, however, is also under control of the cold-induced increase in negative supercoiling of the genomic DNA. The latter mechanism controls cold-induced transcription of genes for RNA chaperons, translation, cell wall metabolism, and regulation of the membrane fluidity.

As mentioned above (section 2.4.1), during perception of the cold stress signal, the sensory histidine kinase Hik33 feels rigidification of the membrane. Hik33 controls only 23 of the 48 highly cold-inducible genes, and the activation mechanism for other cold-inducible genes remained unclear. The cluster analysis of the results obtained with DNA microarrays revealed that expression of the majority of the cold-inducible genes depend on cold-induced increase in negative supercoiling of the genomic DNA (Figure 8). At least, cold-induced transcription of *crhR* for RNA helicase and *rbpA1* for RNA-binding protein could be prevented by the inhibition of the DNA gyrase.

It is well known that acclimation to low temperatures require expression of genes for ROS inactivation, for ribosomal proteins (excess of which compensates for a drop in translational speed at low temperatures), for RNA chaperons (to maintain the RNA matrices unwound), for cell wall and lipid metabolism (to protect the destruction of cell walls and to maintain the fluidity membranes under certain level, which prevents cold-induced lipid phase separation) [2].

All these genes necessary for acclimatization to low temperatures are activated during the cold-induced changes in genomic DNA supercoiling.

## 3. Conclusions and Perspectives

Substantial progress has been made in five recent years in studying and understanding the mechanisms of stress responses in cyanobacteria. That is mainly due to the availability of the data on complete genomes of several cyanobacterial species and due to the development of DNA microarray technique and proteome analysis.

The genome-based systematic analysis provides a powerful technique, which allows to identify the Hiks and Rres that are involved in the perception and transduction of stress signals. Such an approach revealed that Hik33 regulates the expression of most cold-inducible genes in *Synechocystis* and that five two-component systems are involved in the perception of salt stress and hyperosmotic stress, as well as the transduction of the respective signals.

It still remains to be determined how a single two-component system can perceive and transduce more than one kind of environmental signal [28,29]. For example, Hik33-Rre31 is involved in the sensing of salt stress and hyperosmotic stress, but it regulates different sets of genes in response to each respective stress. Similarly, Hik34-Rre1 is involved in the sensing of salt stress and of hyperosmotic stress but also regulates different sets of genes in response to each type of stress. Moreover, Hik33-Rre26 contributes to the regulation of gene expression upon exposure of cells to cold stress and light stress, whereas Hik33-Rre31 does so upon exposure of cells to hyperosmotic and salt stress. It is interesting to note, in this context, that three genes homologous to *hik33*, *rre26,* and *rre31*, respectively, are encoded by the plastid genome of some red and golden-brown algae [132].

The observations, summarized above, cannot be simply explained by the current model of two-component systems, in which a Hik perceives a specific stress and regulates the expression of a particular set of genes via the phosphorylation-dependent activation (or inactivation) of its cognate Rre. A full explanation will require elucidation of more details regarding the mechanisms of signal perception and transduction. It is possible that as-yet-unidentified components are important in determining the specificity of responses to individual types of stress. It is also possible that sensors of environmental signals are highly organized protein complexes, in which Hiks, Rres and various unidentified components are somehow associated and that changes in the composition of the complex could provide specificity.

It is also important to determine the exact fuction of Ser/Thr kinases, sigma factors of RNA polymerases and transcription factors. A combination of physiological, biochemical, and genetic approaches should lead to a deeper understanding of the mechanisms of stress responses in lower and higher organisms.
